# *Origanum vulgare* L. Essential Oil Mitigates Palmitic Acid-Induced Impairments in Insulin Signaling and Glucose Uptake in Human Adipocytes

**DOI:** 10.3390/ph18081128

**Published:** 2025-07-28

**Authors:** Andrea Müller, Jonathan Martinez-Pinto, Claudia Foerster, Mario Díaz-Dosque, Liliam Monsalve, Pedro Cisternas, Barbara Angel, Paulina Ormazabal

**Affiliations:** 1 Institute of Agrifood, Animals and Environmental Sciences, Universidad de O’Higgins, Ruta 90 km 3, San Fernando 3070000, Chile; andrea.muller@uoh.cl (A.M.); claudia.foerster@uoh.cl (C.F.); liliam.monsalve@uoh.cl (L.M.); 2 Instituto de Fisiología, Facultad de Ciencias, Universidad de Valparaíso, Gran Bretaña 1111, Valparaíso 2360102, Chile; jonathan.martinez@uv.cl; 3 Institute for Research in Dental Sciences (ICOD), Faculty of Dentistry, University of Chile, Olivos 943, Independencia, Santiago 8380544, Chile; mrdiaz@uchile.cl; 4 Núcleo de Investigación en Nutrición y Ciencias Alimentarias (NINCAL), Facultad de Salud y Ciencias Sociales, Universidad de las Américas, Av. Manuel Montt 948, Providencia, Santiago 7500658, Chile; pcisternasf@udla.cl; 5 Escuela de Obstetricia, Facultad de Ciencias para el Cuidado de la Salud, Universidad San Sebastián, Lota 2465, Providencia, Santiago 7510157, Chile; barbara.angel@uss.cl

**Keywords:** adipocyte, palmitic acid, insulin signaling, glucose uptake, *Origanum vulgare* L.

## Abstract

**Background**: Obesity is associated with insulin resistance (IR) and characterized by impaired activation of the PI3K/AKT route and glucose uptake. Elevated plasma levels of palmitic acid (PA) diminish insulin signaling in vitro and in vivo. *Origanum vulgare* L. essential oil (OVEO) is rich in monoterpenes with protective effects against IR. **Objective:** The study aimed to assess total phenols content and antioxidant activity of OVEO and its cytotoxicity, as well as its effect on insulin signaling and glucose uptake in PA-treated adipocytes. **Methods**: The quantification of total phenolic content was determined using the Folin–Ciocalteu method, while the antioxidant capacity of OVEO was assessed by DPPH (2,2-diphenyl-1-picrylhydrazyl) and FRAP (ferric reducing antioxidant power) methods. The cytotoxicity of OVEO (0.1–10 µg/mL) was assessed using the MTS assay. SW872 adipocytes were incubated with 0.4 mM PA for 24 h, with or without a 2 h preincubation of OVEO, and then stimulated with insulin (100 nM, 10 min) or a vehicle. Phosphorylation of Tyr-IRS-1, Ser-AKT, and Thr-AS160 was analyzed by Western blot, and glucose uptake was measured using 2-NBDG. **Results**: OVEO contained phenols and exhibits antioxidant capacity. All the concentrations of OVEO assessed were not cytotoxic on SW872 adipocytes. PA decreased basal phospho-AS160 as well as insulin-stimulated phospho-IRS1, phospho-AKT, phospho-AS160 and glucose uptake, while OVEO co-treatment enhanced these markers. **Conclusions**: These findings suggest a beneficial effect of OVEO on the PA-impaired insulin pathway and glucose uptake, which might be explained by its phenolic content and antioxidant capacity, highlighting its potential as a complementary therapeutic agent for IR and related metabolic disorders.

## 1. Introduction

According to the World Health Organization (WHO), between 1990 and 2022, childhood and adolescent obesity increased fivefold, while adult obesity quadrupled. Childhood obesity is associated with a higher risk of developing type 2 diabetes mellitus (T2DM) and cardiovascular diseases, whereas adult obesity is linked to increased mortality and morbidity from various serious health conditions [1,2].

Energy is primarily stored in white adipose tissue (WAT) in the form of triglycerides. During obesity, visceral adiposity impairs insulin signaling, contributing to insulin resistance (IR) and the development of metabolic syndrome. This impairment results from defective activation of the insulin receptor pathway and its downstream substrates IRS-1, AKT, and AS160, ultimately leading to reduced insulin-stimulated glucose uptake in adipocytes [3].

Palmitic acid (PA), a 16-carbon saturated fatty acid, is elevated in individuals with obesity and is linked to metabolic conditions such as nonalcoholic steatohepatitis and IR [4]. In vitro studies have demonstrated that PA-treated adipocytes exhibit reduced insulin-stimulated phosphorylation of IRS-1, AKT, and AS160, along with diminished glucose uptake [5,6]. Natural strategies, including the use of plants and their extracts, have been shown to enhance insulin sensitivity and have attracted considerable interest, particularly due to traditional knowledge regarding their role in the relief and treatment of diseases [7].

*Origanum vulgare* L. (OV), known as oregano, is native to Europe, North Africa, and temperate and tropical regions of Asia, and has been introduced to North and South America. It is an aromatic herb used both as a condiment and as a medicinal plant. The composition of *Origanum vulgare* L. essential oil (OVEO) includes a variety of terpenoids [8]. The concentration of its constituents can vary depending on geographic location, harvest season, genetic variation, the use of fresh or dried plant material, and the specific part of the plant utilized [9]. OV has been reported to exhibit antimicrobial, antioxidant, anti-inflammatory, antitumor, and hypoglycemic activities, among others, both in vitro and in vivo [10]. Evidence from in vitro assays indicates that the aqueous acetonitrile extract of OV can promote glucose uptake, inhibit glycosylation, and relieve oxidative stress when applied to hepatic cells [11], while in vivo, the use of an ethyl acetate OV extract reduced hyperglycemia and inflammation in diabetic mice [12]. However, to our knowledge, there is no evidence regarding the role of OVEO on insulin signaling markers in human adipocytes exposed to saturated fatty acids. In light of this, the present study aimed to investigate the antioxidant potential, polyphenol content, and effects of OVEO on insulin signaling in human adipocytes challenged with PA.

## 2. Results

### 2.1. Chemical Characterization of OVEO

In order to characterize the properties of OVEO, we measured its total phenolic content using the Folin–Ciocalteu method and assessed its antioxidant capacity by the ferric reducing antioxidant power (FRAP) and 2,2-diphenyl-1-picrylhydrazyl (DPPH) methods, as shown in Table 1. For comparative purposes and to provide broader context, data on phenolic content and/or antioxidant capacity from other commonly used medicinal and culinary herbs have also been included in Table 1.

### 2.2. OVEO Exhibits No Cytotoxic Effects on SW872 Adipocytes

**Figure 1:**
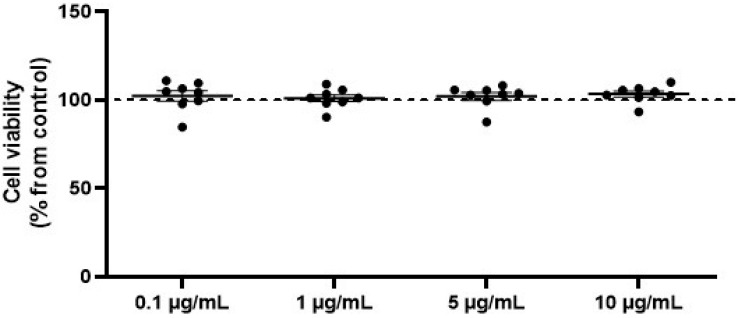
OVEO does not affect viability of SW872 adipocytes. Cells were treated with OVEO (0.1–10 µg/mL, 26 h), and viability was assessed by MTS assay. Results are expressed as % of control (dotted line at 100%). Each dot represents an individual value; the line depicts the mean from eight independent experiments performed in triplicate.

To analyze potential cytotoxicity, SW872 adipocytes were treated with varying concentrations of OVEO (0.1, 1, 5, and 10 µg/mL) for 26 h prior to performing the MTS assay [3-(4,5-dimethylthiazol-2-yl)-5-(3-carboxymethoxyphenyl)-2-(4-sulfophenyl)-2*H*-tetrazolium]. OVEO had no effect on cell viability (Figure 1) at any of the tested concentrations compared to the control. Thus, the experiments were conducted using the lowest OVEO concentration (0.1 µg/mL), as the main focus of this study was to evaluate biological effects at low doses. For reference, the results obtained with OVEO at 10 µg/mL are presented separately in the Supplementary Materials (Figures S1 and S2).

### 2.3. OVEO Restores PA-Impaired AS160 Basal Phosphorylation in SW872 Cells

**Figure 2:**
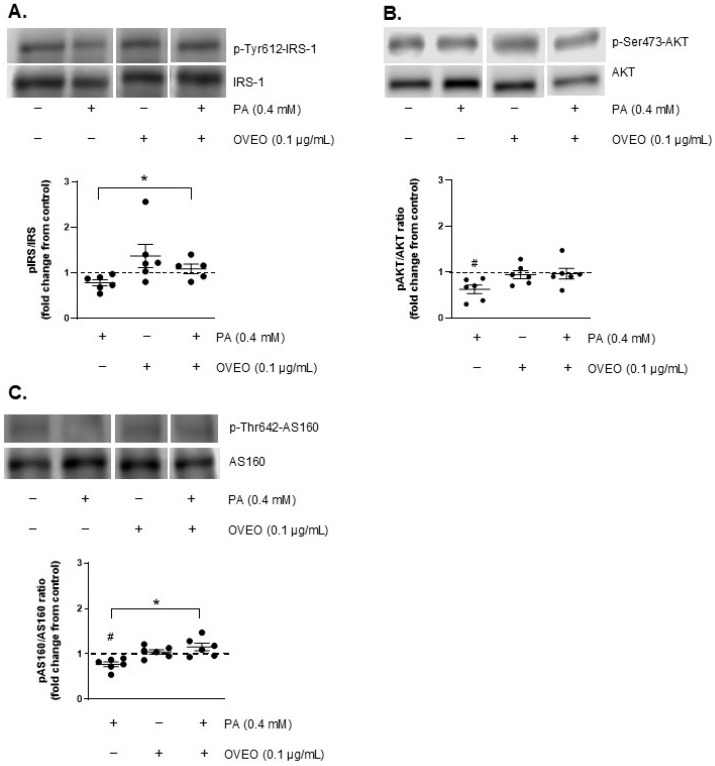
OVEO reverses PA-induced reduction in basal AS160 phosphorylation in SW872 adipocytes. Cells were treated with PA (0.4 mM, 24 h) with or without OVEO (0.1 µg/mL, 26 h). Immunoblots show the basal phosphorylation of (**A**) IRS-1, (**B**) AKT, and (**C**) AS160. Samples were run on the same gel (see Figures S3–S5). Results are presented as fold change relative to control (dotted line). Dots represents individual values; lines depicts the mean from five to six independent experiments. # *p* < 0.05 vs. vehicle; * *p* < 0.05 vs. PA. Analysis was performed using a *t*-test or a generalized linear model, as appropriate.

The impact of OVEO on the basal phosphorylation levels of IRS-1, AKT, and AS160 was evaluated in PA-treated SW872 adipocytes. As shown in Figure 2, treatment with PA reduced the basal phosphorylation of AKT by 37.7% compared to control cells (*p* < 0.05, Figure 2B), and decreased AS160 phosphorylation by 23% relative to untreated cells (*p* < 0.05, Figure 2C). Interestingly, the presence of OVEO in PA-treated cells increased basal p-AS160 levels by 49.6% compared to cells exposed to PA alone (*p* < 0.05, Figure 2C). OVEO did not alter the basal phosphorylation levels of IRS-1, AKT, or AS160 compared to the control cells (Figure 2A–C).

### 2.4. OVEO Restores PA-Impaired IRS-1, AKT, and AS160 Insulin-Stimulated Phosphorylation in SW872 Cells

**Figure 3:**
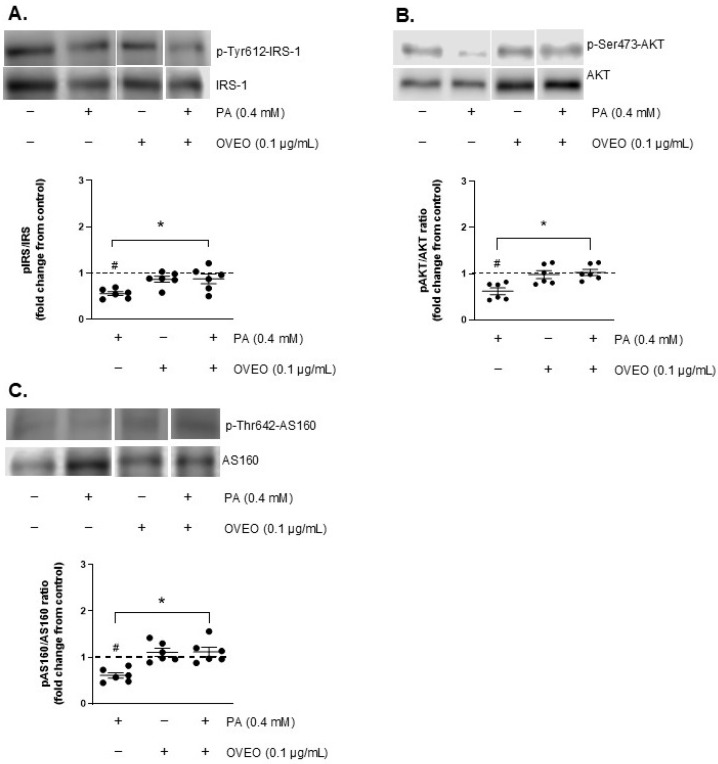
OVEO restores insulin-stimulated phosphorylation of IRS-1, AKT, and AS160 impaired by PA in SW872 adipocytes. Cells were treated with PA (0.4 mM, 24 h) with or without OVEO (0.1 µg/mL, 26 h), followed by insulin (100 nM, 10 min). Immunoblots show the insulin-stimulated phosphorylation of (**A**) IRS-1, (**B**) AKT, and (**C**) AS160. Samples were run on the same gel (see Figures S3–S5). Results are presented as fold change relative to the control (dotted line). Dots represent individual values; lines depict the mean from six independent experiments. # *p* < 0.05 vs. vehicle; * *p* < 0.05 vs. PA. Analysis was performed using a *t*-test or a generalized linear model, as appropriate.

To investigate the effect of OVEO on insulin signaling activation in SW872 adipocytes, the phosphorylation levels of IRS-1, AKT, and AS160 were analyzed. As illustrated in Figure 3A, PA exposure led to a decrease in insulin-induced IRS-1 phosphorylation by 44.5% compared to the control cells (*p* < 0.05, Figure 3A) and in AKT phosphorylation by 38% relative to the control (*p* < 0.05, Figure 3B). PA also reduced p-AS160 levels by 39% compared to the control cells (*p* < 0.05, Figure 3C).

To determine whether OVEO could counteract PA-induced disruption of insulin signaling, we measured p-IRS-1, p-AKT, and p-AS160 levels in adipocytes co-treated with OVEO. For this purpose, OVEO (0.1 µg/mL) was added 2 h before PA treatment and remained present throughout the entire 24 h PA exposure period. The presence of OVEO increased the phosphorylation of IRS-1, AKT, and AS160 by 57.4%, 65.8%, and 83.3%, respectively, compared to PA-treated cells (*p* < 0.05, Figure 3A–C), suggesting that the essential oil can counteract the alterations caused by PA. In contrast, OVEO alone did not modify insulin-stimulated phosphorylation of IRS-1, AKT, or AS160 compared to vehicle-treated controls (Figure 3A–C).

### 2.5. OVEO Counteracts Palmitic Acid-Impaired Glucose Uptake in SW872 Adipocytes

**Figure 4:**
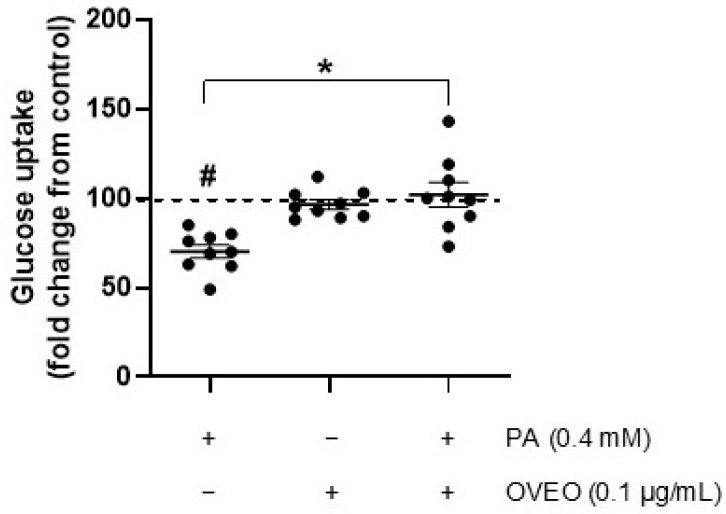
OVEO reverses PA-induced reduction in glucose uptake in SW872 adipocytes. Cells were treated with PA (0.4 mM, 24 h) with or without OVEO (0.1 µg/mL, 26 h), followed by insulin (100 nM, 10 min). Glucose uptake was assessed using 2-(*N*-(7-nitrobenz-2-oxa-1,3-diazol-4-yl)amino)-2-deoxyglucose (2-NBDG). Results are presented as fold change relative to the control (dotted line). Dots represent individual values; lines depict the mean from nine independent experiments. # *p* < 0.05 vs. vehicle; * *p* < 0.05 vs. PA. Analysis was performed using a *t*-test or a generalized linear model, as appropriate.

Since OVEO counteracts the PA-induced reduction in insulin-stimulated phosphorylation of IRS-1, AKT, and AS160 in SW872 adipocytes—and considering that glucose uptake is a key downstream event in insulin signaling—we investigated whether OVEO influences this process in PA-treated cells following insulin stimulation. As expected, PA treatment resulted in a reduction in insulin-induced glucose uptake by 70.2% compared to control cells (*p* < 0.05, Figure 4). Interestingly, the presence of OVEO in PA-treated cells increased glucose uptake to 102.1% (*p* < 0.05, Figure 4). Relative to vehicle-treated cells, OVEO alone did not alter insulin-induced glucose uptake (Figure 4).

## 3. Discussion

Obesity results from complex interactions among biological, genetic, environmental, and behavioral factors, significantly reducing life expectancy. Central adipose tissue accumulation triggers IR, which is a key pathophysiological factor in the development of T2DM and other comorbidities linked to excessive fat accumulation [2,16]. Recent evidence has demonstrated that medicinal compounds derived from plants have the potential to prevent and treat various conditions, including IR associated with obesity and T2DM [7,17]. Herbs have been used for remedial purposes since ancient times, and the WHO estimates that approximately 60% of people use herbal medicine [18]. Origanum extract has been effectively employed as an herbal medicine for the treatment of metabolic conditions [10,19]. The efficacy of herbal preparations is likely attributable to the synergistic interplay among several constituents, rather than the action of a single compound [20]. Accordingly, we focused on evaluating OVEO as a complete mixture rather than isolating and examining its individual pure compounds.

The main components present in OVEO have been identified through chemical characterization by gas chromatography–mass spectrometry, revealing that oxygenated monoterpenes are the primary constituents of the essential oil, followed by monoterpene hydrocarbons and sesquiterpenes [21]. Natural monoterpenes and essential oils rich in these compounds have demonstrated antioxidant activity [22]. Similarly, polyphenols are widely recognized for their antioxidant capacity, both in vitro and in vivo. These bioactive molecules have been shown to ameliorate IR by reducing postprandial glucose levels, modulating glucose transport, and influencing insulin signaling pathways, among other mechanisms [23]. OVEO has been reported to contain phenols and exhibit antioxidant properties. In this study, OVEO extracted from leaves demonstrated excellent antioxidant activity, as evidenced by IC50 values, compared to essential oils from *Poliomintha longiflora* (Mexican oregano) [15] and OV leaves and flowers [24]. The antioxidant potential of OVEO, determined by FRAP, was nearly equal to that of another OV subspecies (Greek oregano, *O. vulgare* L. subsp. *hirtum* (Link) Ietswaart) [25]. Similarly, the phenolic content of OVEO closely approximated the TPC of *Rosmarinus officinalis*, another widely used culinary and medicinal herb [14]. It is important to clarify that the comparison of OVEO with the essential oils of *Rosmarinus officinalis* and Egyptian *Origanum vulgare* in this study was intended solely as a reference to other commonly used medicinal and culinary herbs. As phytochemical equivalence cannot be assumed between these species due to differences in chemotype, geographic origin, and processing methods, such comparisons should be interpreted with caution. This perspective is supported by recent findings from Khademi Doozakhdarreh et al. [26] who demonstrated that the essential oil composition, antioxidant activity, and total phenolic content of *Rosmarinus officinalis* vary significantly depending on harvesting time and drying method. This highlights how environmental and technical factors can markedly influence the chemical profile and biological properties of essential oils, reinforcing the need to interpret interspecies or interstudy comparisons within these limitations. Accordingly, the results presented in this study for OVEO reflect the specific chemotype and experimental conditions under which the oil was obtained and analyzed, including its geographic origin (Chile), extraction process, and preparation method.

Given the presence of phenolic compounds and the antioxidant capacity of OVEO, it was postulated that OVEO could be a promising candidate for in vitro evaluation of its potential to ameliorate impaired insulin signaling in adipocytes. Origanum has been reported to improve IR and modulate the expression of genes involved in carbohydrate metabolism [10]. Interestingly, extracts of OV have demonstrated the ability to improve serum glucose profiles in hyperglycemic and diabetic animal models [27]. Conversely, PA, which is elevated in individuals with obesity, has been linked to IR [4]. This in vitro study is the first to demonstrate the effect of OV on PA-treated SW872 adipocytes. Our findings indicate that OVEO did not affect the viability of SW872 cells and counteracted PA-impaired IRS-1/AKT/AS160 activation and glucose uptake. Evidence from a mouse model of cognitive deficit induced by a high-fat diet (HFD) has shown that thymol, one of the main components of OV [21], enhances the expression of phospho-Ser473 AKT in the brain [28]. Our data revealed that OVEO improved phospho-Ser473 AKT levels in PA-treated adipocytes compared to cells treated only with PA. Therefore, a beneficial role was also evidenced in a human adipose cell line, aligning with the documented protective effects of OVEO’s main constituents against molecular impairments found in models of insulin signaling alteration [28].

Monoterpenes are volatile compounds abundantly present in citrus fruits, vegetables, spices, and herbs. These compounds are classified as terpenes consisting of two isoprene units and may exhibit either acyclic or cyclic structures [22]. Some of the abundant terpenes present in OVEO include cis-sabinene hydrate, 4-terpineol, thymol, and γ-terpinene, among others that are less represented in the essential oil [21]. On the other hand, glucose uptake is crucial for regulating plasma glucose levels, thereby directly influencing glucose tolerance [3]. Notably, monoterpenes such as limonene have been demonstrated to enhance glucose uptake in 3T3-L1 adipocytes [29]. Our findings indicate an improvement in PA-impaired glucose uptake in SW872 adipocytes treated with OVEO, which might be related to its monoterpene content. Nevertheless, additional research is required to determine the precise relationship between specific components of the monoterpene family in OVEO and glucose uptake.

In this study, PA exposure reduced insulin-stimulated phosphorylation of IRS-1, AKT, and AS160, along with impairing glucose uptake in SW872 adipocytes. Interestingly, these impairments in phosphorylation and glucose uptake were abrogated by OVEO. Unfortunately, we did not find scientific evidence on the effects of OVEO’s most abundant compounds on these specific phosphorylation events. However, data are available on the pathophysiological phenomena associated with IR and T2DM. Terpineol, one of the main constituents of OVEO [21], has been shown to enhance insulin sensitivity in rats fed an HFD [30]. Thymol, a natural phenolic monoterpenoid found in OVEO [21], reduced body weight gain, hemoglobin A1C, and glucose levels, as well as reversed peripheral insulin resistance in an obese murine model fed an HFD [28,31]. Although we did not evaluate OVEO’s effects on obesity-related disruptions in animal models, OVEO exhibited a protective effect by alleviating the molecular alterations caused by PA in SW872 adipocytes.

Carvacrol, a natural liquid phenolic monoterpenoid found in the essential oil of OV, has been shown to influence the PI3K/AKT pathway. In streptozotocin-induced type 1 diabetes mellitus and T2DM db/db mice, intraperitoneal administration of carvacrol exhibited anti-diabetic effects. Specifically, carvacrol significantly improved blood glucose levels, increased the phosphorylation of 3-phosphoinositide-dependent protein kinase-1 (PDK1) and phosphoinositide 3-kinase (PI3K), and decreased the phosphorylation of phosphatase and tensin homolog (PTEN) [32]. As a constituent of OVEO, carvacrol may contribute to the observed effects; however, it cannot be excluded that these effects result from the synergistic action of carvacrol with other more abundant components in the essential oil. Further studies are required to elucidate the specific contributions of these compounds.

**Figure 5:**
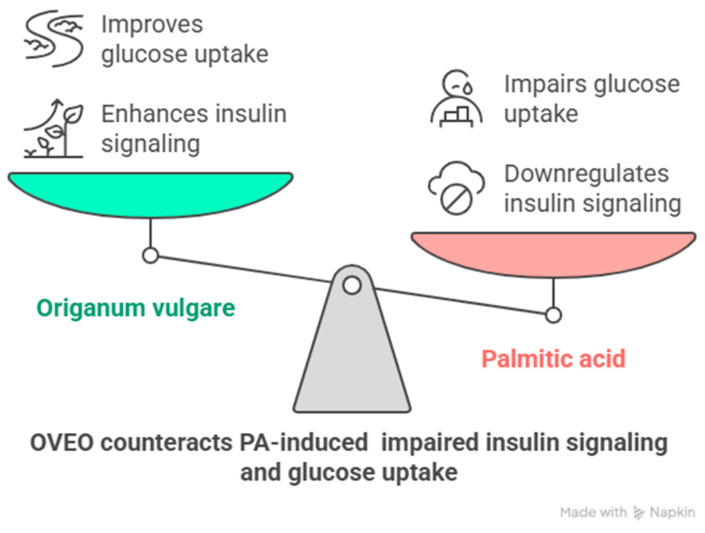
Schematic representation of main findings of the role of OVEO on PA-treated SW872 adipocytes. Image created with Napkin.AI beta 2025.

In summary, *Origanum vulgare* exhibits antioxidant activity both in vitro and in vivo [10]. Similarly, polyphenols and terpenes are well known for their ability to improve insulin responsiveness in these settings [22,23]. In this study, OVEO counteracted PA-induced downregulation of IRS-1, AKT, and AS160 activation, as well as glucose uptake, in SW872 adipocytes (Figure 5). These findings may be attributed to the phytochemical content of OVEO.

## 4. Materials and Methods

### 4.1. Herb Material and Origanum vulgare L. Essential Oil (OVEO)

Aerial parts of *Origanum vulgare* L. were harvested during the flowering stage in Chicauma, Lampa, Metropolitan Region, Chile (33°14′23″ S, 70°54′27″ W). Botanical identification was performed by the taxonomist Alicia Marticorena (MSc) at the CONC Herbarium, Universidad de Concepción, Chile (specimen ID: CONC 191040). Essential oil was obtained from 300 g of fresh leaves through steam distillation for 3 h, yielding approximately 2.5 mL per cycle. The oil’s density was determined to be 0.920 g/mL, based on the mass ratio of 1 mL of essential oil to 1 mL of distilled water at 20 °C. The extracted oil was stored at −20 °C until further use.

### 4.2. Total Phenolic Content (TPC)

The content of total polyphenols present in the OVEO sample was assessed by the Folin–Ciocalteu spectrophotometric method according to Santos et al. [33] with some modifications. A volume of 210 µL of distilled water was placed into 96-well plates. Then, 30 µL of sample, 80 µL of 20% sodium carbonate, and 100 µL of Folin–Ciocalteu reagent (Sigma Aldrich, Taufkirchen, Germany) were added to each well. The reaction mixture was incubated at room temperature for 60 min in the dark, and absorbance was subsequently measured at 765 nm using a microplate reader (Epoch, Biotek Instruments, Winooski, VT, USA). Gallic acid (GA; Sigma Aldrich, Taufkirchen, Germany) served as the calibration standard, and results were reported as milligrams of gallic acid equivalents per gram (mg GAE/g).

### 4.3. Antioxidant Power

The antioxidant capacity of OVEO was determined by DPPH and FRAP spectrophotometric methods. An aliquot of the OVEO solution (50 µL) was mixed with 250 µL of 0.5 mM DPPH (Sigma Aldrich, Taufkirchen, Germany) solution. The plate was incubated for 30 min at room temperature and in the dark. Absorbance was read at 517 nm using an Infinite F50 reader (Tecan^®^, Männedorf, Switzerland). Trolox was used as a standard solution (5–100 ppm). The percentage of inhibition was determined using the following equation:

$$\frac{\left(Acontrol-Asample\right)}{Acontrol}\times 100$$

where Acontrol is the absorbance of DPPH and Asample is the absorbance of the reference standard and extract, respectively. The plot of % inhibition versus concentration was then generated, and the IC_50_ (mean inhibitory concentration) in mg/L was calculated.

For the FRAP assay, a sample of OVEO (20 µL) was mixed with 180 µL of FRAP solution (consisting of 300 mM acetate buffer, 10 mM 2,4,6-tri(2-pyridyl)-s-triazine (TPTZ), and 20 mM FeCl3). The mixture was incubated at 37 °C for 15 min. After incubation, absorbance was read at 593 nm using an Infinite F50 reader (Tecan^®^). Trolox (Sigma Aldrich, Taufkirchen, Germany) (µmol TEAC/g) and ascorbic acid (Sigma Aldrich, Taufkirchen, Germany) (mg AAE/g) were used as standard solutions.

### 4.4. SW872 Cell Culture and Adipogenic Differentiation

The SW872 cell line (ATCC HTB-92, Manassas, VA, USA), originally derived from a human fibrosarcoma, was used in this study. Preadipocytes were maintained in a humidified incubator at 37 °C with 5% CO_2_ and cultured in Dulbecco’s modified Eagle’s medium/nutrient mixture F-12 (DMEM/F-12, Sigma-Aldrich, St. Louis, MO, USA) supplemented with 10% fetal bovine serum (FBS, Biological Industries, Beit-Haemek, Israel) and antibiotics (penicillin–streptomycin, Biological Industries). Upon reaching confluence, differentiation was induced by culturing the cells for 10 days in DMEM/F-12 with 1% FBS, supplemented with 1 M dexamethasone (Sigma-Aldrich), 1 M rosiglitazone (Calbiochem^®^, Darmstadt, Germany), 10 μg/mL insulin (Insuman^®^, Sanofi-Aventis, Paris, France), and 0.5 mM 1-methyl-3-isobutylxanthine (Calbiochem^®^). Maturation into adipocytes was confirmed by the accumulation of lipid droplets [6].

### 4.5. Assessment of Cell Viability and Experimental Treatments

Adipocyte viability following exposure to various concentrations of OVEO was assessed using the CellTiter 96^®^ Aqueous One Solution Cell Proliferation Assay (Promega, Madison, WI, USA), in accordance with the manufacturer’s protocol. This assay was employed to determine the appropriate OVEO concentration for subsequent experiments. After differentiation (day 10), cells were incubated with 0.1, 1.0, 5, and 10 µg/mL of OVEO. The incubation period with OVEO was 26 h, corresponding to the maximum exposure time used in this study, which included 24 h of co-treatment with OVEO + PA plus 2 h of pre-treatment with OVEO. Following treatments, 20 µL of MTS reagent were added. After 3 h of incubation, absorbance at 490 nm was measured using an ELx808 microplate reader (BioTek Instruments, Inc., Winooski, VT, USA). Background absorbance at 630 nm was recorded and subtracted from the A490 readings. Data were expressed as a percentage relative to the control group.

**Figure 6:**
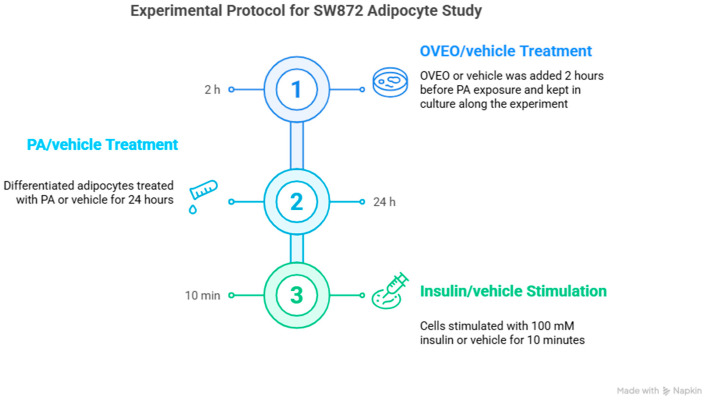
Schematic representation of treatments in differentiated SW872 adipocytes. Image created with Napkin.AI beta 2025.

A 100 mM PA (Sigma-Aldrich, St. Louis, MO, USA) stock solution was prepared by dissolving PA in 0.1 M NaOH and heating at 70 °C in a shaking water bath. In parallel, a 10% fatty acid–free bovine serum albumin (FFA-BSA; Sigma-Aldrich) solution was prepared in water at 55 °C. The PA solution was then conjugated to the BSA solution by continuous stirring, according to the method presented by Cousin et al. [34], before dilution in the culture medium. Following the completion of the differentiation process, SW872 adipocytes were incubated with PA or vehicle (0.1 M NaOH and FFA-BSA solution) for 24 h with or without OVEO (2 h preincubation) and then stimulated with insulin (100 mM, 10 min) or vehicle (Figure 6). Therefore, the experimental conditions were as follows: untreated cells (control), 0.4 mM PA, 0.1 µg/mL OVEO, or 0.1 µg/mL OVEO (2 h prior) + 0.4 mM PA, under insulin-stimulated or basal conditions. The insulin concentration and exposure duration were selected based on prior studies demonstrating a two-fold increase in AKT phosphorylation relative to unstimulated adipocytes [35].

### 4.6. Western Blot Analysis

Total protein lysates were prepared by sonicating SW872 cells at 4 °C in a lysis buffer containing 50 mM Tris base, 150 mM NaCl, 10 mM sodium pyrophosphate, 100 mM NaF, 2 mM orthovanadate, and 1% NP-40 (pH 8.0), supplemented with PhosSTOP (Roche, Mannheim, Germany). Protein concentrations were determined using the bicinchoninic acid assay (Pierce, Rockford, IL, USA). Samples containing 40 µg of total protein were heat-denatured in sodium dodecyl sulfate–polyacrylamide gel electrophoresis (SDS-PAGE) loading buffer (240 mM Tris-HCl, 40% glycerol, 8% SDS, 20% 2-mercaptoethanol, pH 6.8). Proteins were separated on 8–10% polyacrylamide gels and transferred onto 0.22 µm nitrocellulose membranes (Amersham™ Protran^®^, Munich, Germany) using a cold transfer buffer (194 mM glycine, 24 mM Tris, 20% methanol). Membranes were blocked with 5% BSA in Tris-buffered saline containing 0.05% Tween-20 (TBS-T), and then incubated with the following primary antibodies: anti-IRS-1 (E-12) (1:300; Santa Cruz Biotechnology, Dallas, TX, USA), phospho-IRS-1 (Tyr612) (1:500; Invitrogen, Carlsbad, CA, USA), anti-AKT (1:1000; Cell Signaling, Danvers, MA, USA), phospho-AKT (Ser473) (1:1000; Cell Signaling), anti-AS160 (C69A7) (1:500; Cell Signaling), and phospho-AS160 (Thr642) (1:500; Cell Signaling). β-actin (1:3000; Santa Cruz Biotechnology) was used as the internal loading control.

Following incubation with peroxidase-linked secondary antibodies, immune complexes were visualized using the substrate Westar Supernova (Cyanagen, BO, Italy), and detection was performed with the LI-COR C-DiGit Blot Scanner (Lincoln, NE, USA). Band intensities were quantified using ImageJ software version 1.53h (NIH, Bethesda, MD, USA). Densitometric comparisons were made using bands derived from the same membrane.

### 4.7. Glucose Uptake

To assess glucose uptake, equivalent quantities of SW872 preadipocytes were plated in 6-well dishes, and upon reaching confluence, the cells underwent differentiation as described above. Following a 10-day differentiation period, adipocytes were exposed or not to 0.4 mM PA for 24 h, with or without OVEO, as previously described. After treatment, cells were washed and incubated in serum-free DMEM (Sigma-Aldrich, St. Louis, MO, USA) for 2 h. The medium was then removed, and cells were rinsed twice with glucose-free Kreb’s Ringer buffer (KRB) containing 145 mM NaCl, 10 mM HEPES (pH 7.4), 2.6 mM CaCl_2_, 5 mM KCl, and 1 mM MgCl_2_. Next, adipocytes were stimulated with 100 mM insulin in glucose-free KRB for 30 min at 37 °C under 5% CO_2_. Afterwards, the fluorescent glucose analog 2-NBDG (Cayman Chemical, Ann Arbor, MI, USA) was added, and cells were incubated for an additional 15 min. Uptake was halted by aspirating the medium, and cells were washed twice with glucose-free KRB. Lysis was performed by adding a buffer composed of 150 mM NaCl, 50 mM Tris base, and 1% NP-40 (pH 8.0) directly to the wells. Lysates were transferred to black, clear-bottom 96-well plates, and fluorescence was measured at 465/540 nm (excitation/emission) using a Synergy 2 fluorimeter (BioTek Instruments Inc., Winooski, VT, USA).

### 4.8. Statistical Analysis

Normality of data distribution was evaluated using the Shapiro–Wilk test. Comparisons between two experimental conditions were conducted using Student’s *t*-test. Statistical analyses were performed with STATA software, version 18.8. Data are presented as dot plots indicating individual values, with the mean represented by a horizontal line. A *p*-value below 0.05 was considered statistically significant.

## 5. Conclusions

OVEO mitigated PA-impaired insulin-stimulated activation of IRS-1, AKT, and AS160, as well as glucose uptake in human SW872 adipocytes. We suggest that the phenolic and terpene content of OVEO contributes to its impact on PA-treated adipocytes. However, additional research is required to clarify the role of OVEO in modulating insulin sensitivity disrupted by saturated fatty acids, a phenomenon observed in obesity and other metabolic conditions. Future research—especially in vivo studies—is needed to evaluate the efficacy and underlying biological mechanisms of OVEO in counteracting insulin resistance. Moreover, examining the bioavailability of its constituents, along with their safety and effectiveness in human populations, will be essential to support the potential use of OVEO as a therapeutic option for obesity-associated disorders.

## Figures and Tables

**Table 1:** Total phenols quantification and antioxidant activity of *Origanum vulgare* L. essential oil and other species.

Sample	TPC (mg GAE/g)	DPPH (mg/L)	DPPH IC_50_ (mg/L)	FRAP (mg AAE/g)
*Origanum vulgare* (OVEO)	11.85 ± 0.29 ^1^	8.09 ± 0.27 ^1^	57.58 ± 0.30 ^1^	10.83 ± 0.42 ^1^
*Rosmarinus officinalis* (rosemary) [13,14]	13.77 ± 1.30 ^2^	6.00 ± 0.10 ^1^	-	-
*Poliomintha longiflora* (Mexican oregano) [15]	27.85 ± 0.15 ^1^	-	83.70 ± 4.12 ^1^	-

Data is shown as means with standard deviations. ^1^ Data obtained from an essential oil of the species. ^2^ Data obtained from an acetone/perchloric acid extract of the species. DPPH: DPPH scavenging capacity; DPPH IC_50_: concentration of the sample required to inhibit 50% of radical; FRAP: ferric reducing antioxidant; TPC: total phenolic content; GAE: gallic acid equivalents; AAE: ascorbic acid equivalents.

## Data Availability

Data will be made available upon reasonable request due to institutional restrictions.

## Supplementary Materials

The following supporting information can be downloaded at: https://www.mdpi.com/article/10.3390/ph18081128/s1, Figure S1: Effect of 10 μg/mL OVEO on basal phosphorylation of IRS-1, AKT, and AS160 in PA-treated SW872 adipocytes. Figure S2: Effect of 10 μg/mL OVEO on insulin-stimulated phosphorylation of IRS-1, AKT, and AS160 in PA-treated SW872 adipocytes. Figure S3: Full image of the blot for phospho-IRS-1 and IRS-1 in basal (non-insulin-stimulated) and insulin-stimulated SW872 adipocytes; Figure S4: Full image of the blot for phospho-AKT and AKT in basal (non-insulin-stimulated) and insulin-stimulated SW872 adipocytes; Figure S5: Full image of the blot for phospho-AS160 and AS160 in basal (non-insulin-stimulated) and insulin-stimulated SW872 adipocytes.

## Abbreviations

The following abbreviations are used in this manuscript:

AAE	Ascorbic acid equivalent
BSA	Bovine serum albumin
DMEM	Dulbecco’s modified Eagle’s medium
DPPH	2,2-Diphenyl-1-picrylhydrazyl
FBS	Fetal bovine serum
FFA	Fatty acid-free
FRAP	Ferric reducing antioxidant power
GA	Gallic acid
GAE	Gallic acid equivalents
HFD	High-fat diet
IC_50_	Mean inhibitory concentration
IR	Insulin resistance
KRB	Kreb’s Ringer buffer
MTS	3-(4,5-Dimethylthiazol-2-yl)-5-(3-carboxymethoxyphenyl)-2-(4-sulfophenyl)-2*H*-tetrazolium
2-NBDG	2-(*N*-(7-Nitrobenz-2-oxa-1,3-diazol-4-yl)Amino)-2-Deoxyglucose
OV	*Origanum vulgare*
OVEO	*Origanum vulgare* essential oil
PA	Palmitic acid
PAGE	Polyacrylamide gel electrophoresis
PDK1	3-phosphoinositide-dependent protein kinase-1
PI3K	Phosphoinositide 3-kinase
PTEN	Phosphatase and tensin homolog
T2DM	Type-2 diabetes mellitus
TBS-T	Tris-buffered saline containing 0.05% Tween-20
TEAC	Trolox equivalent antioxidant capacity
TPC	Total phenolic content
TPTZ	2,4,6-tri(2-pyridyl)-s-triazine
WAT	White adipose tissue
WHO	World Health Organization
