# Supplementary figures and images for: Origanum vulgare L. Essential Oil Mitigates Palmitic Acid-Induced Impairments in Insulin Signaling and Glucose Uptake in Human Adipocytes
Source: Pharmaceuticals (Basel). 2025 Jul 28;18(8):1128. doi: 10.3390/ph18081128 (PMC12389695; doi:10.3390/ph18081128)

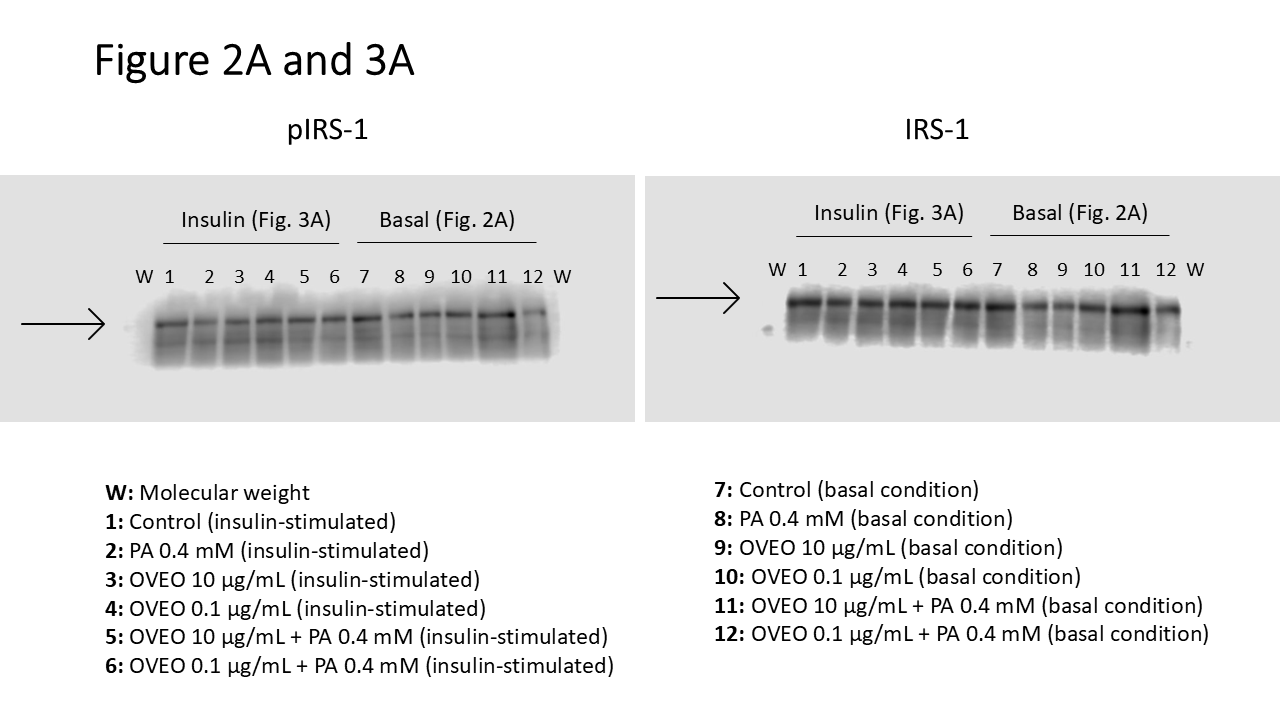

Supplement: Supplementary file 1 [file pharmaceuticals-18-01128-s001.zip › Figure S3_WB Fig 2A and 3A pIRS & IRS.tif]

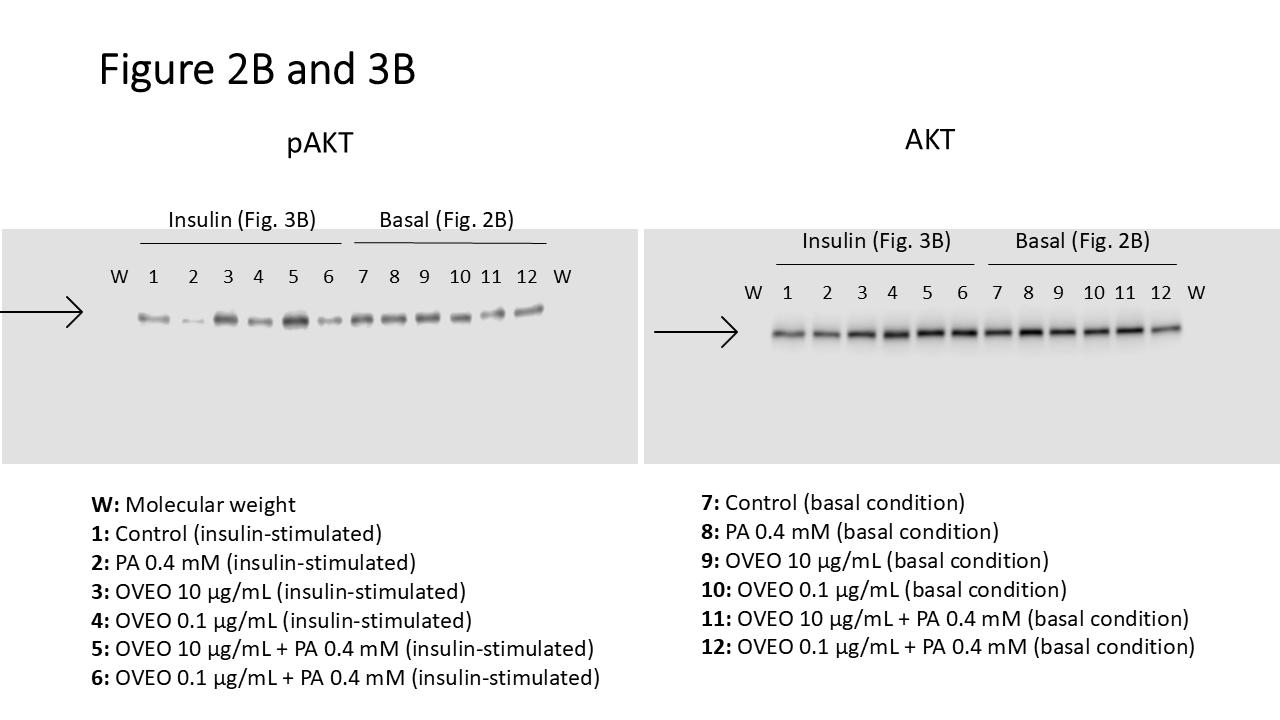

Supplement: Supplementary file 1 [file pharmaceuticals-18-01128-s001.zip › Figure S4_WB Fig 2B and 3B pAKT & AKT.tif]

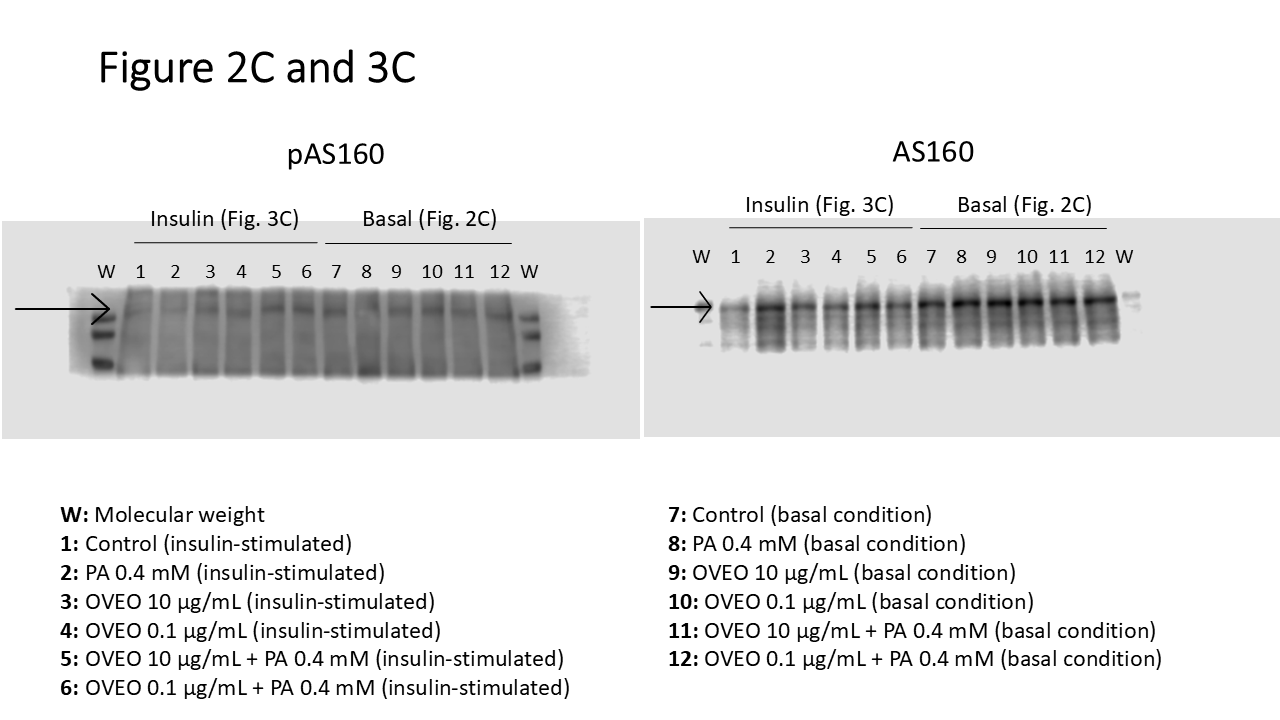

Supplement: Supplementary file 1 [file pharmaceuticals-18-01128-s001.zip › Figure S5_WB Fig 2C and 3C pAS160 & AS160.tif]

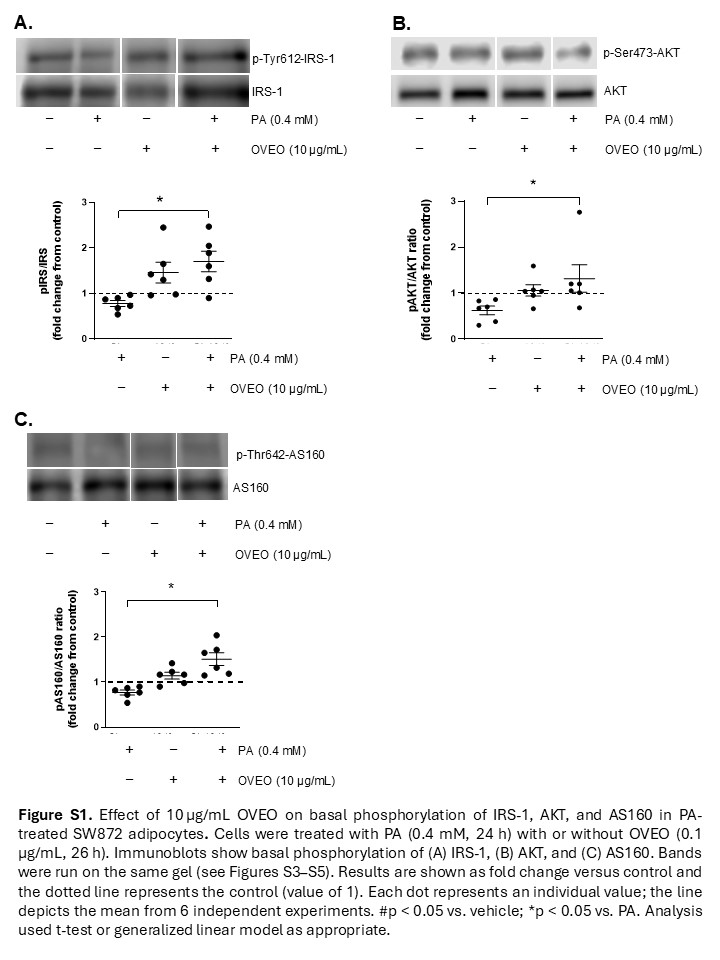

Supplement: Supplementary file 1 [file pharmaceuticals-18-01128-s001.zip › Figure_S1.JPG]

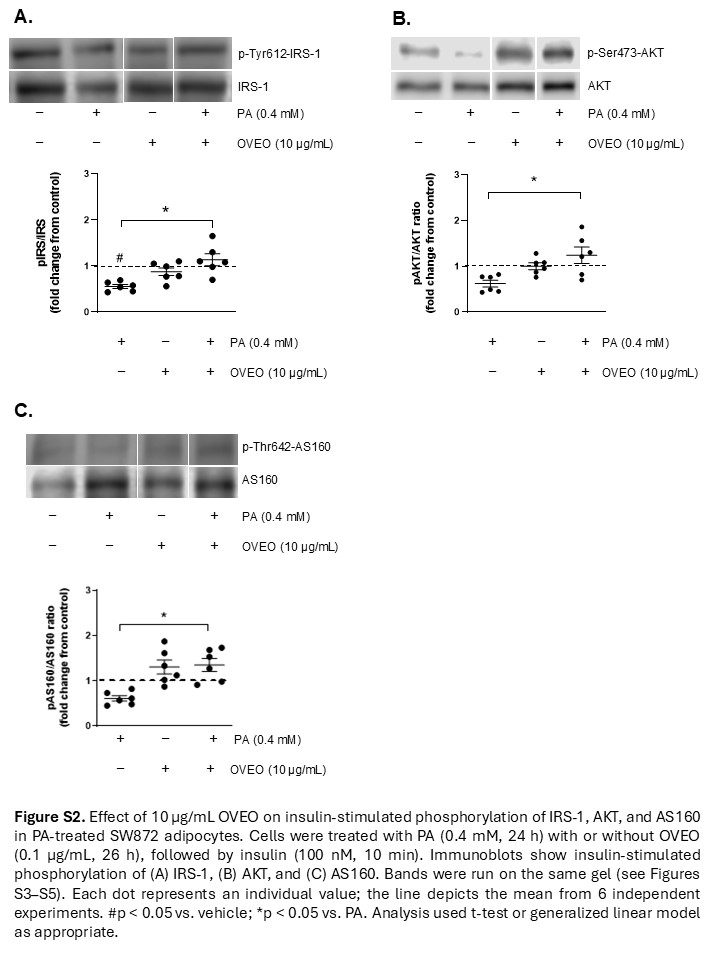

Supplement: Supplementary file 1 [file pharmaceuticals-18-01128-s001.zip › Figure_S2.JPG]
